# Long-Term Complication of Three-Level Cervical Artificial Total Disc Replacement: A Case Report

**DOI:** 10.7759/cureus.42380

**Published:** 2023-07-24

**Authors:** Ganesh Phayal, Amrit Chiluwal, Salvatore M Zavarella

**Affiliations:** 1 Neurological Surgery, SpineCare Long Island, Long Island, USA; 2 Neurological Surgery, College of Medicine, State University of New York (SUNY) Downstate Health Sciences University, Brooklyn, USA; 3 Neurosurgery, SpineCare Long Island, Long Island, USA

**Keywords:** residual exposed endplate, dysphagia requiring surgical relief, cervical artificial total disc replacement, hybrid surgery, osteolysis, heterotopic ossification, paracervical pain, cervical degenerative disc disease, anterior cervical discectomy and fusion

## Abstract

Anterior cervical discectomy and fusion (ACDF) has long been the standard surgical treatment for cervical degenerative disc disease (DDD); however, cervical artificial total disc replacement (cTDR) has gained increasing recognition in recent years due to its ability to maintain a natural range of motion and lower the rate of adjacent segment disease. Although cTDR is only approved for one or two levels in the United States, it has been used for three or more levels in other countries. We present a case of a 59-year-old male patient who underwent three-level cTDR (C4-C7) in Germany and presented 10 years later with progressive paracervical pain and worsening dysphagia. Magnetic resonance imaging (MRI) and computed tomography (CT) scan showed hardware loosening, progressive loss of bone around the device, and a cyst ventral to C4-C5 with mass effect on the hypopharynx. The patient was successfully treated with posterior cervical fusion and showed improvement in neck pain. This case underscores the significance of long-term follow-up and thoughtful consideration when selecting an appropriate treatment modality for patients afflicted with cervical DDD.

## Introduction

Cervical degenerative disc disease (DDD) is a commonly encountered condition in aging patients presenting with a spectrum of symptoms including neck pain, cervical radiculopathy, and myelopathy. In addition to these, patients may also suffer from other neurological symptoms and severe pain, which may require multimodal management. Initial management is usually conservative, including oral analgesics, physical therapy, or interventional pain procedures. Surgical intervention is required in cases where the patient is myelopathic or unresponsive to conservative management [1]. The traditional surgical approach to treat cervical DDD is anterior cervical discectomy and fusion (ACDF), which involves anterior decompression of the disc space followed by interbody grafting and fusion [2]. Anterior cervical fusion procedure was first described by Smith and Robinson in 1958 and since then the number of such procedures performed in the US is rapidly increasing. This procedure has been widely adopted due to its efficacy with minimal complications, including rare complications such as post-operative dysphagia, hematoma, recurrent laryngeal nerve palsy, and esophageal perforation. Esophageal perforation is primarily associated with high mortality. However, mortality and morbidity remain very low at 0.1% and 20%, respectively, which signifies the excellent outcomes of ACDF. This type of surgery can also be performed in an ambulatory surgery center in selected patients who are younger than 65 years old, which makes this procedure easily accessible and efficient. However, a significant drawback of fusion surgery is the reduced range of motion post-operatively [3-4]. An alternative approach to ACDF is cervical artificial total disc replacement (cTDR), which allows for maintaining a natural range of motion, pseudoarthrosis, and dysphagia, and lowers the risk of adjacent-level bone degeneration [5]. In the US, only one or two level-cTDR is approved by the FDA for certain devices. PRESTIGE ST, PRODISC-C, BRYAN, SECURE-C, PCM, Mobi-C, PRESTIGE LP, M6-C, and Simplify are approved for single-level cTDR, whereas Mob-C and PRESTIGE LP are approved for two-level procedure as well [6]. This report describes a rare case of a 59-year-old male patient, who underwent a cervical cTDR of C4-C7 in Germany and presented 10 years later with progressive paracervical pain and worsening dysphagia.

## Case presentation

History and examination

**Figure 1:**
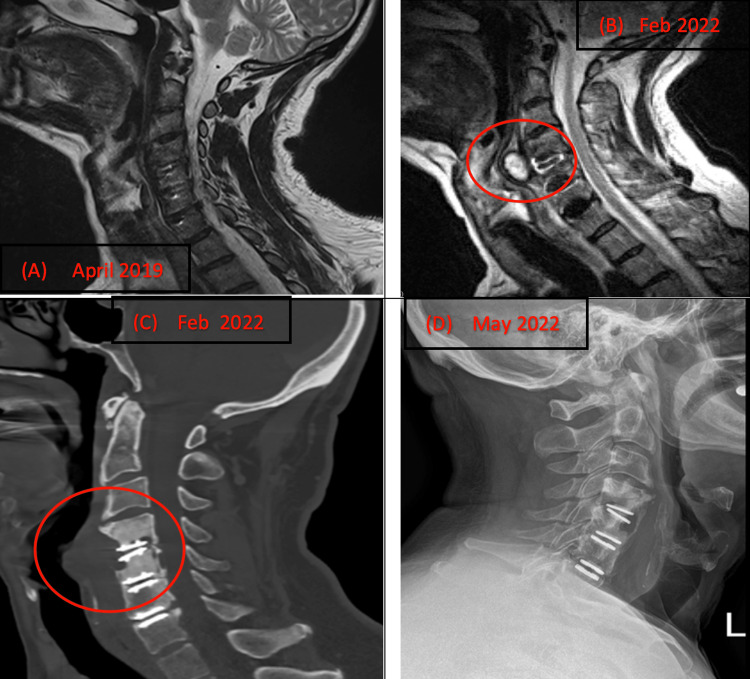
Images showing progressive changes seen within three years. (A) MRI of C4-C5 level shows sclerosis; (B) Cyst (red circle) is seen centrally anterior to the C4-C5 measuring 2.1 cm x 2.7 cm x 1.7 cm, right paracentral to the C4-C5 measuring 2.1 cm x 1.2 cm x 1.2 cm and left paracentral to the C3-C4 measuring 2.3 cm x 1.2 cm x 1.1 cm (not shown in the figure). (C) Mass effect on hypopharynx that is anteriorly displaced (red circle). Prevertebral soft tissue abnormality measuring 5.3 cm x 2.1 cm x 5.2 cm at the C4-C6 level.* *(D) X-ray: Osteolysis at the C4-C5 level with progressive loosening of the disc replacement hardware and progressive loss of bone around the devices at C4-C5 and to a lesser degree at C5-C6.

A 59-year-old male patient visited our office due to progressive paracervical pain, which was not radicular, and radiated to both shoulders but not to the arms. He reported no weakness in his upper extremities, and the motor strength of his deltoid, biceps, triceps, wrist flexion and extension, intrinsics, hip flexion, knee flexion and extension, dorsiflexion, and plantarflexion was all 5/5. The patient's reflexes, including biceps, triceps, brachioradialis, patellar, Achilles, Hoffman, and Babinski reflexes, were all 2+. Cranial nerves II-XII were also intact. The patient demonstrated normal gait and had no issues during the finger-to-nose testing, heel-to-shin testing, and pronator drift. The patient had undergone a three-level total disc replacement at C4-C7 10 years ago in Germany, and a one-level lumbar spine disc replacement in the past. The initial CT scan showed C5-C7 total disc replacement that had progressed to fusion around the disc replacement. Nonetheless, there was significant remodeling and sclerosis of bones at the C4-C5 level that had caused cavitation within the C5 body. A subsequent MRI with and without contrast revealed underlying sclerosis, but there was no significant neural compression (Figure 1A).

After two years, the patient returned to our office with severe paracervical pain spreading to his shoulders and difficulty swallowing. An MRI revealed a large cyst that was moderately heterogeneous and hyperintense located ventral to the C4-C5 disc replacement (Figure 1B). The mass was found to be compressing the esophagus (Figure 1C). A biopsy confirmed that there was no underlying infection. A follow-up MRI, CT scans, and X-rays indicated significant osteolysis at the C4-C5 level with progressive loosening of the disc replacement hardware and a progressive loss of bone around the devices at C4-C5, and to a lesser degree, at C5-C6 (Figure 1D). As a result, we recommended surgery involving posterior C4-C7 decompression, stabilization, and fusion.

Treatment and management

**Figure 2:**
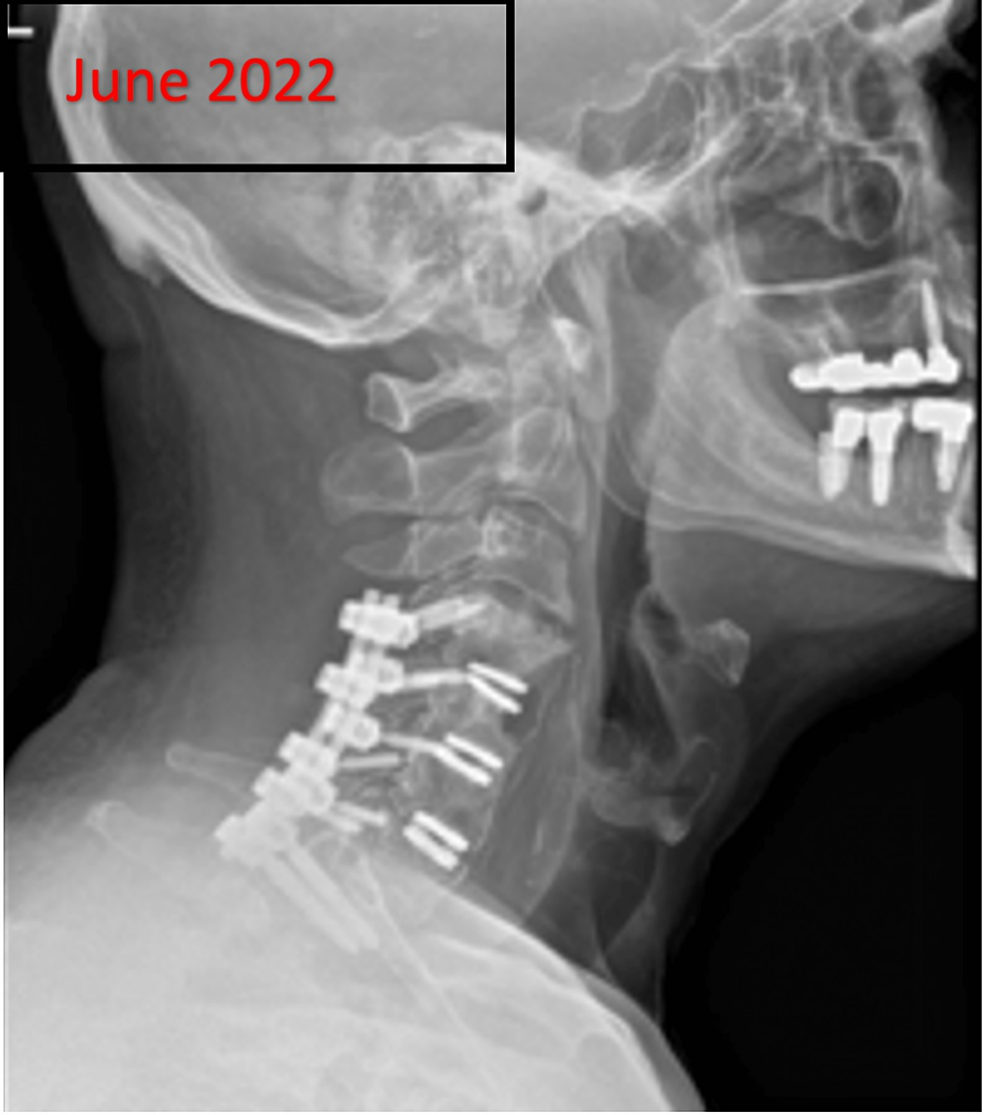
X-ray: Post-operative-Anterior discectomy at C4-C5, C5-C6 and C6-C7. The visualized soft tissues reveal no abnormalities.

The recommended course of action was fusion surgery to reduce the abnormal motion across C4-C7 disc replacement spacers. This involved performing a C4-C7 lateral mass screw fixation with fusion, utilizing MAGNUS allograft, ACTIFUSE allograft, and harvested autograft. The surgical procedure was closely monitored using fluoroscopy, somatosensory evoked potential (SSEP), and electromyography (EMG). To ensure a successful outcome, antibiotics were used for incision irrigation. The neurosurgical aspect of the surgery was completed without any issues (Figure 2). Following the surgery, the patient was doing well and was advised to undergo physical therapy while wearing a Miami J cervical collar for the next eight weeks.

## Discussion

In this report, we presented a case of a patient who had undergone a three-level artificial disc replacement at the C4-C7 level in Germany and returned with severe and progressive paracervical pain 10 years later. The FDA recommendation in the United States limits the use of cTDR for up to two levels with a specific prosthesis, unlike in Europe and Asia. Only nine artificial discs have been approved by the FDA for single-level procedures, and three of them for two-level procedures. Proper device selection is critical to achieving positive postoperative outcomes, as certain devices have been associated with complications. Devices like PRESTIGE ST are associated with an increased incidence of dysphagia and hoarseness; hence, the device has been removed from the US market [6-8]. While ACDF is the traditional approach for treating cervical DDD, cTDR is an alternative procedure that can preserve motion and lower reoperation rates. Younger patients and those with demanding lifestyles often prefer cTDR over ACDF. However, there is a lack of evidence for three or more levels of cTDR, which may be a significant barrier to its use in every case.

Although Heterotopic ossification (HO) is not prevalent in the early postoperative period, studies suggest that long-term HO is highly associated with cTDR, regardless of sex or age [9]. HO can negate the benefits of motion preservation from the cTDR procedure. According to McAfee classification, HO is graded from low to high, with high-grade HO associated with an increased residual exposed endplate (REE). An REE greater than 2 mm increases the chance of developing grade three or four HO, which accelerates the degeneration of adjacent segments. Although inferior-segment degeneration rates are equally prevalent in both cTDR and ACDF cases, superior-segment degeneration rates are not [9, 10]. Some studies have found that HO is less prevalent in multilevel cTDR than in single-level procedures at a two-year follow-up, while others have found no significant differences [11, 12]. Further investigation is needed to determine whether HO occurrence is due to cTDR or the differences in the patient’s pathology and/or follow-up period.

When treating multilevel degenerative disc disease (DDD), hybrid surgery can be considered as an alternative to using either cTDR or ACDF in isolation. Hybrid surgery combines cTDR and ACDF procedures at multiple levels in a single operation. Although there are no discernible differences between hybrid surgery and cTDR or ACDF alone in terms of early postoperative benefits or complications such as unintended intubation, pneumonia, or readmission rates, further research is needed to determine whether the same holds true for long-term postoperative outcomes [8].

Our case is unique because the patient did not experience any perioperative or postoperative complications for 10 years. However, 10 years after the initial surgery, the patient reported paracervical pain without any neurological deficits. Over time, the pain worsened, and between the 10th and 12th year, the patient started experiencing progressive dysphagic symptoms due to soft tissue growth ventral to the C4-C5 level. This case emphasizes the importance of considering the long-term effects of multilevel cTDR. While previous studies have demonstrated the benefits of range of motion (ROM) preservation and reduced reoperation rates with the use of cTDR, these benefits become negligible if significant HO occurs. Previous studies have shown that the incidence of HO after cTDR has no clinical relevance during a two- or five-year follow-up period, highlighting the need for longer follow-up periods, such as the 10 years period as presented in our case [13, 14]. The subpopulation typically selected for cTDR procedures is younger (under 65 years old) with increased lifestyle demands, raising concerns about the durability of cTDR in younger patients with longer life expectancies. Therefore, more evidence is needed to support the long-term safety and efficacy of cTDR as a viable option for multilevel cervical procedures.

## Conclusions

We present the case of a 59-year-old man who experienced worsening paracervical pain a decade after a three-level cTDR procedure performed in Germany, as the US FDA does not recommend cTDR beyond one or two levels. Over time, the patient's pain became more severe, and he developed dysphagia due to a cyst near the C4-C5 level. The patient was ultimately treated with a posterior cervical fusion procedure and has since made a good recovery. This case underscores the potential for long-term complications associated with multilevel cTDR procedures and highlights the importance of careful consideration when choosing a treatment option for patients. Including this case report in the current literature may help guide clinicians in making informed decisions when managing similar cases.
